# Partnering with health system operations leadership to develop a controlled implementation trial

**DOI:** 10.1186/s13012-016-0385-7

**Published:** 2016-02-24

**Authors:** Mark S. Bauer, Christopher Miller, Bo Kim, Robert Lew, Kendra Weaver, Craig Coldwell, Kathy Henderson, Sally Holmes, Marjorie Nealon Seibert, Kelly Stolzmann, A. Rani Elwy, JoAnn Kirchner

**Affiliations:** 1 VA Boston Healthcare System, Harvard Medical School, 150 South Huntington Avenue (152M), Boston, MA 02130 USA; 2 VA Boston Healthcare System, Boston University School of Medicine, 150 South Huntington Avenue (MAVERIC), Boston, MA 02130 USA; 3 James H. Quillen VA Medical Center, Corner of Lamont & Veterans Way, Mountain Home, TN 37684 USA; 4 VA New England Healthcare System, Boston University School of Medicine, 200 Springs Road, Building 61 (136G), Bedford, MA 01730 USA; 5 Central Arkansas Veterans Healthcare System, University of Arkansas for Medical Sciences, 2200 Fort Roots Drive, Building 58, North Little Rock, AR 72114 USA; 6 VA Boston Healthcare System, 150 South Huntington Avenue (152M), Boston, MA 02130 USA

**Keywords:** Cooperative behavior, Health-care quality, access, and evaluation, Health plan implementation, Mental disorders, Patient care management, Patient satisfaction, Randomized controlled trial, Self care

## Abstract

**Background:**

Outcome for mental health conditions is suboptimal, and care is fragmented. Evidence from controlled trials indicates that collaborative chronic care models (CCMs) can improve outcomes in a broad array of mental health conditions. US Department of Veterans Affairs leadership launched a nationwide initiative to establish multidisciplinary teams in general mental health clinics in all medical centers. As part of this effort, leadership partnered with implementation researchers to develop a program evaluation protocol to provide rigorous scientific data to address two implementation questions: (1) Can evidence-based CCMs be successfully implemented using existing staff in general mental health clinics supported by internal and external implementation facilitation? (2) What is the impact of CCM implementation efforts on patient health status and perceptions of care?

**Methods/design:**

Health system operation leaders and researchers partnered in an iterative process to design a protocol that balances operational priorities, scientific rigor, and feasibility. Joint design decisions addressed identification of study sites, patient population of interest, intervention design, and outcome assessment and analysis. Nine sites have been enrolled in the intervention-implementation hybrid type III stepped-wedge design. Using balanced randomization, sites have been assigned to receive implementation support in one of three waves beginning at 4-month intervals, with support lasting 12 months. Implementation support consists of US Center for Disease Control’s Replicating Effective Programs strategy supplemented by external and internal implementation facilitation support and is compared to dissemination of materials plus technical assistance conference calls. Formative evaluation focuses on the recipients, context, innovation, and facilitation process. Summative evaluation combines quantitative and qualitative outcomes. Quantitative CCM fidelity measures (at the site level) plus health outcome measures (at the patient level; *n* = 765) are collected in a repeated measures design and analyzed with general linear modeling. Qualitative data from provider interviews at baseline and 1 year elaborate CCM fidelity data and provide insights into barriers and facilitators of implementation.

**Discussion:**

Conducting a jointly designed, highly controlled protocol in the context of health system operational priorities increases the likelihood that time-sensitive questions of operational importance will be answered rigorously and that the outcomes will result in sustainable change in the health-care system.

**Trial registration:**

NCT02543840 (https://www.clinicaltrials.gov/ct2/show/NCT02543840).

## Background

### Collaborative chronic care models and mental health outcome

Mental health conditions affect 46.6 % of Americans during their lives and impact 26.6 % in any given year [1]. Outcome for mental health conditions is suboptimal, and care coordination is problematic, even in integrated health-care systems like the US Department of Veterans Affairs (VA) [2, 3]. Multicomponent care models that emphasize care coordination and evidence-based care have been shown to improve health outcomes for individuals across a variety of mental health conditions.

Specifically, collaborative chronic care models (CCMs) were initially articulated by Wagner and colleagues [4, 5] and elaborated as part of the Robert Wood Johnson Improving Chronic Illness Care initiative [6]. CCMs were initially developed for chronic medical illnesses, stimulated by the recognition that single-component interventions were insufficient to improve outcome in such conditions [4, 5]. CCMs consist of several or all of six components:

- Work role redesign to support anticipatory, continuous care;
- Patient self-management support;
- Provider decision support through simplified practice guidelines and/or facilitated access to specialty consultation;
- Use of clinical information systems for panel management and provider feedback;
- Linkage to community resources; and
- Health care leadership and organization support [4, 5, 7–9].

Examples of how CCM elements can be operationalized in practice are provided in Table 1.

**Table 1:** Examples of operationalization of the CCM

CCM goal: anticipatory, continuous, evidence-based, collaborative care via…				
Work role redesign	Self-management support for individuals in treatment	Decision support	Information management	Community linkages
• Care management • Access-driven scheduling • Activated follow-up	• Incorporation of the individual’s values and skills • Shared decision-making • Self-management skiIIs • Behavioral change interventions	• Provider education • Practice guidelines • Specialty consultation	• *Population*: registry • *Provider*: reminders • Outcome tracking • Feedback • Integrated care plans	• Additional resources • Peer-based support
Organizational leadership and support				

Multiple randomized controlled trials have shown that CCMs improve outcomes for various chronic medical illnesses [7–9] and depression treated in primary care [10, 11]. CCM principles have informed patient-centered medical homes [12] as well as VA primary care-mental health integration efforts [13].

Additional work has extended CCM application to a variety of chronic mental health conditions treated in mental health clinics, with an evidence base for some complex conditions like bipolar disorder that is sufficient to warrant endorsement in national practice guidelines [14, 15] and listing on the SAMHSA National Registry of Evidence-Based Programs and Practices [16]. Overall, meta-analytic work indicates that CCMs have robust effects in a variety of mental health conditions and across both primary care and specialty care settings at no net cost [17, 18].

### CCM implementation challenges and opportunities

However, innovations such as CCMs do not naturally diffuse into common practice, even in integrated health-care systems. For example, after highly successful randomized clinical trials of the CCM for bipolar disorder in two integrated health-care systems, despite designing the studies from the outset as effectiveness trials [19, 20], no participating site continued the model after the trial ended. Thus, not surprisingly, specific efforts are needed to move innovations into sustainable practice [21, 22].

The opportunity to implement CCMs on a system-wide, potentially sustainable basis developed when the VA Office of Mental Health Operations (OMHO) began a high priority effort to enhance care coordination in general mental health clinics by establishing multidisciplinary teams in every VA medical center. Beginning in 2013, this Behavioral Health Interdisciplinary Program (BHIP) initiative directed facilities to develop teams to provide continuous access to recovery-oriented, evidence-based treatment, emphasizing population-based care, consistent with the VA’s *Handbook on Uniform Mental Health Services in Medical Centers and Clinics* [23]. BHIP teams provide multidisciplinary care guided by a staffing model of 5–7 full-time equivalent staff caring for a panel of 1000 patients. Facilities are provided centralized guidance [24] to institute care processes that are consistent with broad BHIP principles, but they are given broad latitude to develop team practices based on local priorities, resources, and conditions. The advantage to this flexible approach is that individual facilities have latitude to respond to local conditions in pursuing national goals; however, the challenge is that while the overall goals are clear, there is no certainty that facilities will employ evidence-based care processes.

In 2014, OMHO leaders partnered with implementation researchers to review the evidence base for team-based mental health care, and in 2015, OMHO endorsed the CCM as the model to inform BHIP team formation. The partnership obtained funding through a national competitive program evaluation process sponsored by the VA Quality Enhancement Research Initiative (QUERI) [25] to conduct a randomized quality improvement program evaluation to investigate two overarching propositions: (a) BHIPs can demonstrate impact on patient health status by incorporating elements of the evidence-based CCM and (b) focused implementation support is needed to support local efforts to establish such teams.

The protocol responds to time-sensitive health system needs, with design elements collaboratively developed to balance operational priorities, scientific rigor, and real-world feasibility. This protocol is described in the next section, with further description of specific partnered design decisions found in the “Discussion” section.

## Methods/design

### Implementation models and evaluation proposals

We designed a hybrid type III implementation-effectiveness controlled program evaluation [26] in order to investigate both implementation and health outcomes in the context of implementing an innovation with established evidence. Notably, this project relies on existing facility staff rather than incorporating exogenous research-funded staff as has been typical in traditional randomized controlled trials.

We chose an evidence-based implementation framework based on the US Center for Disease Control’s Replicating Effective Programs [27], augmented with internal and external implementation facilitation support [28] (called REP-F), which we have previously used jointly to implement the CCM in publicly funded health centers [29]. Analysis of the implementation effort is guided by the Integrated Promoting Action Research on Implementation in Health Services (i-PARIHS) framework, which proposes that successful implementation is a function of facilitation of an innovation with recipients who are supported and constrained within an inner and outer context [30].

We specifically hypothesize that, compared to technical assistance plus dissemination of CCM materials, REP-F-based implementation to establish CCM-based BHIPs will result in

H1a: increased veteran perceptions of CCM-based care,

H1b: higher rates of achieving national CCM fidelity measures, and

H1c: higher provider ratings of the presence of CCM elements (implementation outcomes), as well as

H2: improved veteran health status compared to BHIPs supported by dissemination material alone (intervention outcomes).

**Fig. 1:**
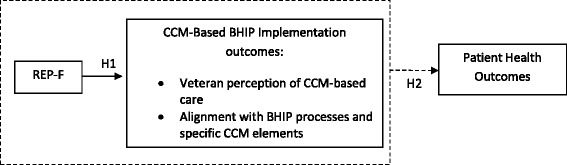
We hypothesize that REP-F implementation support will enhance the establishment of CCM processes within the BHIP teams (H1), which will then result in improved health outcomes for patients (H2)

The overall model relating implementation strategy and CCM intervention to outcomes is diagrammed in Fig. 1.

### Stepped-wedge trial design

To investigate these proposals, we utilize a stepped-wedge-controlled trial design. Stepped-wedge designs are randomized incomplete block designs which, though having a long history in scientific research [31, 32], have only recently been applied to controlled trials or program evaluations. Such designs provide the intervention of interest (REP-F in this protocol) to all participants, but stagger the timing of introduction [33–36]. The stepped-wedge design is increasingly used where all participants must receive the intervention for policy or ethical reasons [36] and has recently been used for CCM implementation in primary care [33]. In the current project, the stepped-wedge design confers two benefits: we can (a) extend implementation support to the maximal number of facilities and (b) enhance information from the formative evaluation of our implementation process.

**Fig. 2:**
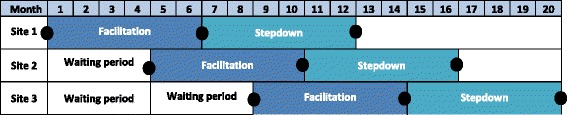
This figure illustrates the stepped wedge for one of the three external facilitators, who will work with three facilities over the course of the study. *Black dots* represent times of health status assessment for patients. Provider interviews and administrative data measure collection occur at the beginning of implementation and at the end of the step-down period

We are utilizing a nested design, randomizing at the site level while using individual veterans as the unit of observation for primary quantitative outcome measures. Based on power calculations (see below), nine sites have been randomly assigned to receive REP-F support in one of three waves beginning at 4-month intervals, with REP-F support lasting 12 months (Fig. 2). The initial phase of REP-F implementation support lasts 6 months. In the second 6 months, the three sites that received REP-F gradually taper to step-down support (less frequent implementation meetings and consultation to the BHIP team on an as-needed basis). While waiting, sites will receive continued access to the extensive BHIP implementation materials on an internal VA website [24] and regular technical support conference calls, plus distribution of a workbook on incorporating CCM elements into existing BHIP teams.

### Site selection and balance

Operations leaders asked in particular that we work with sites that have requested help to establish a BHIP team. We thus jointly developed these site inclusion criteria:

- Self-identification of desire for assistance in developing a BHIP, as evidenced by invitation from the facility mental health service lead to concurrence of the facility director,
- Prior identification of BHIP team members, and
- Allocation of a staff member with quality improvement experience to serve as internal facilitator for 12 months at 10 % effort.

Facilities were recruited through a process involving cascading publicity from OMHO through the regional Veterans Integrated Service Networks (VISNs) to individual facility mental health service leaders.

Site-based randomization in any health-care system must face the reality that sites will never be completely matched on all relevant site characteristics, measured and unmeasured [37]. We therefore used a restricted selection method of randomization to balance key site characteristics across the three implementation waves [38]. We identified the following relevant site characteristics via OMHO-researcher consensus:

- BHIP penetration rate, from national administrative measures;
- Outpatient mental health service delivery characteristics, including average visits/year and telephonic vs. face-to-face care, from national administrative measures;
- Prior success at the facility level with a mental health system redesign effort (penetration rate of primary care/mental health integration), from a national administrative measure;
- Prior systems redesign experience for outpatient mental health staff, from a national provider survey;
- Organizational climate with regard to civility and psychological safety in outpatient mental health, from a national provider survey;
- Broader facility context including rurality and complexity rating, from national administrative measures; and
- Administrative region (VISN).

We then utilized a computer-based algorithm to balance site characteristics as closely as possible [39, 40] across the three waves. After excluding highly collinear characteristics, the algorithm generated 1680 possible site combinations, and we randomly selected a grouping from 1 % of the best balanced options.

### REP-F implementation procedures

REP-F implementation is deployed across the four REP stages: assessing preconditions, pre-implementation preparation, implementation, and post-implementation maintenance. REP-F is operationalized in this program via the following activities:

- In-depth pre-site visit evaluation and orientation to the facilitation process and the CCM, including surveys and/or telephone-based interviews with facility leadership and mental health service leaders, internal facilitator, BHIP team members, and key stakeholders
- Kick-off site visit of approximately 1.5 days
- Weekly videoconferences with the BHIP team and/or conference calls with the internal facilitator as well as ad hoc telephone and email communications
- Step-down support during the second 6 months of facilitation

**Fig. 3:**
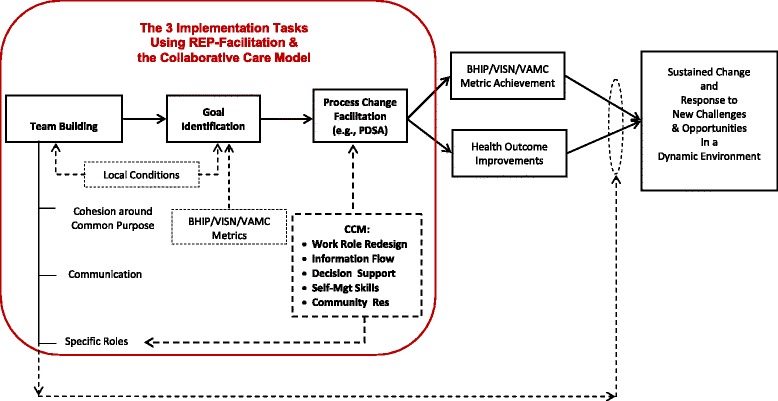
As outlined in the text, this application of REP-F emphasizes the steps of team-building, identification of common goals based on local and national priorities, and process redesign as keys to eventual sustainment of system change. The steps are illustrated sequentially, but the process is iterative and nonlinear [41, 42]

Our pilot work led us to recognize the difference between facilitating to establish a single process or time-limited project and the need to build an ongoing team that can not only establish CCM processes but also adapt them sustainably as local conditions change. We therefore organized our efforts according to three overarching facilitation tasks (Fig. 3):

- Team-building,
- Identification of specific CCM goals and processes based on local conditions, and
- Process change support.

Note, however, that these activities must be considered iterative and not strictly chronological, since implementation progress is likely to be nonlinear [41, 42]. Team-building is a critical step toward change and sustainability, as recognized by complex adaptive system approaches, but not necessarily present in more focused and time-limited QI efforts [41, 42]. Goal-identification is conducted in light of both national BHIP process measures and local priorities. Finally, to achieve those goals, specific process changes must be identified and implemented using traditional quality improvement techniques that empower the team to make ongoing iterative changes to their processes to best adapt as local conditions change, e.g., using plan-do-study-act cycles [43]. We incorporate CCM elements into identifying and changing processes, and this also feeds back on team-building as work roles are redesigned.

### Formative evaluation of the implementation process

We plan our formative evaluation process according to the i-PARIHS update of the original PARIHS model [44], including four types of formative evaluation [45]: developmental, implementation-focused, progress-focused, and interpretive formative evaluation. Developmental formative evaluation will make use of extensive pre-site visit assessment materials, including pre-site visit key informant interviews, meeting with BHIP team members and relevant stakeholders, and review of the site-balancing characteristics enumerated above. Implementation-focused evaluation will focus on methods of operationalizing the CCM elements in a specific medical center. Progress-focused evaluation will make use of multiple sources of input to identify the barriers and facilitators to implementation progress, considering each of the i-PARIHS domains; these sources include regular review of progress toward specified clinical process changes, assessment of team strength, and regular debriefing among internal and external facilitators. Summative evaluation, as outlined in hypotheses 1 and 2, will include both qualitative and quantitative implementation outcomes as detailed below.

### Summative quantitative evaluation (H1a, H1b, H2)

Subject-level measures will be collected in a repeated measures design via telephone interview, at the beginning of implementation and at 6 and 12 months of implementation. Primary quantitative health status outcomes include the Patient Assessment of Chronic Illness Care (PACIC) [46] (H1a), site- and veteran-level indices of BHIP clinical fidelity measures (H1b), and the veteran-level mental component score (MCS) and physical component score (PCS) of the VR-12 [47] (H2). The evaluation is quantitatively powered for H2, specifically the VR-12 MCS (90 % power, alpha = 0.05, effect size = 0.20 [18]). We will also collect patient satisfaction data using the Satisfaction Index [48] and the recovery-oriented Quality of Life Enjoyment and Satisfaction Questionnaire [49] as secondary measures. Note that the sample size accommodates “early looks” at the data at the end of each wave, in order to inform operational partners of emerging results in an operationally relevant time frame.

We are enrolling a survey sample of 85 veterans at each of nine sites (*n* = 765 total) who have had at least two treatment contacts over the prior year in a BHIP clinic. The sole exclusion criterion will be an encounter for dementia within the prior year, since their ability to complete the battery accurately would be questionable. We will gather administrative dataset-based demographic and clinical data on all BHIP veterans through our recruitment procedure and so can identify and adjust for sampling biases and systematic differences across sites and time points. For instance, we can investigate possible population changes over the course of the protocol, e.g., the possibility that as BHIP teams become established, their sicker (or healthier) veterans are referred.

Veteran- and site-level fidelity measures that reflect the CCM (H1b) are being developed nationally by OMHO. These will be collected from operationally gathered performance data. This provides the advantage that we can benchmark all participating sites against national performance rates, ensures relevance of trial outcomes to operational priorities, and decreases project resources for data collection.

Our analytic plan for H2 assesses veteran health status over time within subjects while minimizing respondent burden by using brief telephone interview at baseline and at 6 and 12 months of implementation. The primary evaluation compares the change from baseline to 12 months, the end of the implementation period (the implement and step-down periods in Fig. 1). Secondary evaluations will explore changes from baseline to 6 months and from 6 to 12 months to indicate if the effects tend to appear early or late in the 12-month period. The design allows evaluation of within-site changes in health status and quantitative process outcomes. Also, having staggered implementation but balancing site characteristics over time, we can assess the effects of secular trends at several calendar times with cross-sectional contrasts of a site undergoing implementation with a site awaiting implementation.

Our quantitative analyses will utilize repeated measures mixed effects general linear modeling (GLM) [50–52], with factors of intervention, site, time, and with subject within site as a random effects. GLM quantifies and apportions the variance in outcome (e.g., MCS) among relevant factors, thus isolating the change in outcome due to the primary contrast of interest (in this evaluation, REP-F implementation support vs. baseline). The mixed model accommodates repeated measures (within-subject correlation), random effects (subject), and moderate imbalance among independent factors (sites) and assumes that missing data are missing at random. We will explore results for patterns of unequal variance, relevant correlation structures, and variance component models to ensure that our results are robust. Additionally, the site sample sizes are large enough to explore various site-specific effects by adding site-interaction terms to the model.

Regarding missing data, we will test the robustness of results against nonrandom dropout patterns using Bayesian methods for the pattern mixture model [53, 54]. This systematically models the missing data using intensive Bayesian Monte Carlo Markov chain imputation to explore a wide range of potential non-missing mechanisms. These models for missing data dovetail with the proposed mixed model for the observed data allowing statistical tests of how explicit bias arising from nonrandom missingness alters the results of the primary analysis [54].

Additionally, secondary exploratory analyses will add covariates to determine the degree to which baseline factors explain the overall change. For instance, other independent variables include site characteristics and veteran characteristics such as demographics, mental health diagnoses, and history of mental health hospitalization.

A similar approach will be taken to analyze H1a, which proposes that veteran perceptions of CCM care, as measured by the PACIC, will be greater after REP-F implementation than at baseline. For H1b, we will also analyze those OMHO national BHIP process measures that are amenable to veteran-level measurement, comparing pre- to post-implementation status as above.

We will model response in a logistic regression model overall and by site to profile who responds and who does not using the covariates listed above as well as calendar time and status of implementation (pre or post). During the evaluation, we will also construct a propensity score for response with data from the entire general mental health clinic population at each medical center to predict future response and validate the primary analyses using propensity score weights as applied to clinical trials [55].

### Summative qualitative evaluation (H1c)

Data from qualitative analyses will serve two purposes. First, *directed content analysis* [56] focusing on identification of CCM elements will provide data with which to assess fidelity to the intervention-dependent variable for H1c. Second, *grounded thematic analysis* [57] will contribute to interpretive formative evaluation [45] at the end of the evaluation, which could help explain unexpected implementation findings and refine facilitation steps to use in future efforts.

We will identify and consent four BHIP clinical staff per site, ensuring interdisciplinary representation across physicians, nurses, social workers, and psychologists. Interviews will be conducted via telephone, audio-recorded, and transcribed verbatim. There will be 72 total interviews: nine sites, four providers per site, and each interviewed pre- and post-implementation. Interviews will be de-identified, including information regarding the provider, site, and pre/post-implementation status of each interview.

We have oriented our qualitative analyses to complement our quantitative analyses, planning parallel data collection with integration post hoc [58]. For directed content analysis [56] to identify CCM elements, we will code data relevant to the presence or absence of each of the six CCM elements. Coded material will be summarized in narrative form (using principles of data reduction consistent with Miles and Huberman [57]). Based on coded data and summaries, each site’s fidelity to CCM elements will be rated on a scale of 0–4 to facilitate comparisons across sites and over time.

Our pilot work provided valuable methodologic data for our directed content analyses. We conducted semi-structured qualitative interviews with mental health providers at three medical centers with various levels of BHIP experience. We used an iterative procedure to develop a codebook for assessing the extent to which care at these three sites was consistent with each of the six CCM elements. Our codebook was organized according to the structure described by MacQueen and colleagues [59], featuring both brief and detailed definitions of each of the six CCM elements, as well as guidelines for when to apply (and not apply) each CCM element code, along with examples. We initially aimed to apply this codebook using rapid assessment [60] to identify individual quotes that were indicative of one or more CCM elements. We were unable to obtain adequate inter-rater reliability using this method, however, as we found that decontextualized quotes did not contain sufficient detail to identify CCM elements.

We therefore shifted to an approach in which interviews from each site were analyzed at the site level. We developed a separate site-level narrative that summarized care processes that were consistent (or not) with each of the six CCM elements. Whenever possible, this site narrative referred to specific quotes from the interviews but did not rely solely on such quotes taken out of the context of the individual interviews. We were able to quickly achieve consensus regarding site-level ratings using this method, distinguishing systematic differences among the three sites regarding consistency with CCM principles.

Formal statistical analyses of directed content analysis data are not appropriate [61]. However, our a priori proposal (H1c) is that for each site, CCM scores will increase from pre- to post-implementation, and we will be able to describe the degree to which this occurred within and across sites. Additionally, we will assess individual elements from provider’s ratings in each site to identify patterns of implementation across sites for individual CCM elements, which would add internal consistency validity to our conclusions; that is, common patterns would support (though not prove [56]) generalizability of implementation strategy effects.

For the interpretive formative evaluation [45], we will code interviews using our grounded thematic analysis [57] coding, paying particular attention to factors that might be barriers or facilitators to future implementation efforts [62]. These analyses will also be used to contextualize and explain our directed content analyses above.

### Cost analysis

We will conduct a cost analysis based on time-motion assessments as in our previous work [63]. Specifically, we will choose random weeks to have external facilitators log all implementation-related activities during the first and second 6 months of implementation, including calls, emails, meetings, and product development. This, plus initial site visit time and travel cost, will provide a stable estimate of external facilitation expenses. For each site, we will also estimate the internal facilitator’s time in the same manner and estimate the time spent by clinical and support BHIP team members via scheduling analysis focusing on meetings related to team development (but not clinical activities). This will provide OMHO and facility leaders in the field with a reasonable estimate of the personnel and related costs of this implementation strategy.

### Limitations and anticipated challenges

Despite utilizing pilot funding to make various design decisions in an evidence-based manner, several limitations warrant consideration. First, our clinical intervention is a multicomponent model, the CCM, rather than a single-process intervention. The complex adaptive systems [41, 42] perspective predicts that such a flexibly implemented multicomponent model will have greater success in improving health outcomes than focusing on a single process. While we have considered this carefully both conceptually and operationally, we recognize that the manner in which the six CCM elements will be deployed will be diverse across sites. We have developed a qualitative analysis strategy that will accommodate this diversity by allowing each element to be assessed individually, but in the context of CCM expectations, by directed content analysis. Moreover, we utilize a dual approach to maximize information yield from qualitative data, combining directed content analysis [59, 61] to identify CCM elements with grounded thematic analysis [57] to elucidate key facilitator and barriers to provide data to support OMHO’s plan for subsequent BHIP implementation.

Second, we have designed our quantitative veteran-level health status assessment as a within-subjects design, following individual veterans at three assessments over 12 months. We anticipate the need for replacement as veterans leave care or decline further participation. Based on our extensive experience with long-term clinical trial outcomes monitoring [19, 20], we have designed a very low-burden follow-up assessment procedure to minimize dropout, but plan to replace veterans who drop out and conduct sensitivity analyses to determine the degree to which conclusions are affected by including original participants with those who enter later.

Despite its advantages, the nested stepped-wedge design also has some limitations. As with any site-level intervention, subjects cannot be randomized to intervention or control condition nor can intervention precede nonintervention. Implementation in waves over time introduces possible time trends, and we cannot perfectly balance site characteristics over time, although our design allows us to identify such trends. Clustering and missing values that might produce dropout bias require us to make strong assumptions to analyze the data. These unavoidable issues call for the sensitivity analyses described above.

Finally, as with all hybrid designs, this work requires a multidisciplinary evaluation team. Additionally, the effort requires a multi-faceted project management plan that includes both parallel and serial tasks. The fact that this project is supported by both competitive grant funding and operations support means that each of the study tasks must be completed in the context of an evolving clinical and operational context. Success will require drawing on our diverse experience as operations experts, implementation scientists, clinical trialists, qualitative researchers, and managers of multi-site evaluation projects.

### Trial status

This project includes both a quality improvement program evaluation component and a research component and has been approved as such by the VA Central Institutional Review Board. Specifically, the initiative to implement CCM-based BHIP is considered a quality improvement program evaluation project for which medical center leadership volunteers their facility, and individual consent of providers for this process is not obtained. In contrast, the participation of providers in the qualitative interviews is fully voluntary (and kept confidential from facility leadership) and considered research and therefore subject to informed consent. Similarly, veteran health status and care perception assessment are considered research, and informed consent is obtained. The investigators’ home sites are considered “engaged in research,” while the participating sites are not, since they are identifying the population of providers and veterans from which to recruit but are not themselves recruiting the subjects.

An advisory board has been constituted, including health system operational leaders, researchers, and a veteran representative. It has met regularly to design the protocol and will continue to meet to monitor study conduct and results.

Site recruitment using cascading publicity from OMHO to VISN mental health leads to facility mental health leadership was very successful, within 2 months exceeding the enrollment target of nine facilities. Nine sites have been formally enrolled with three additional sites which volunteered to receive facilitation support outside of the formal trial. At the time of this writing, the first wave of sites has been engaged in REP pre-implementation assessment.

## Discussion

This project results from the collaborative work of health system operations partners and a multidisciplinary group of researchers, supported by competitive funding from the VA’s innovative QUERI program [25]. To review all the substantive discussions and decision-making processes that informed protocol design is precluded by space limitations. Nevertheless, the most salient or widely relevant discussions and design decisions are summarized in Table 2.

**Table 2:** Key partner-based evaluation protocol design decisions

Design element	Operational considerations	Researcher considerations
Sites and population		
The BHIP operational initiative has already begun.	Need for results to inform continuing process. Can capitalize on momentum of the system to engage and motivate sites, promulgate best-practice models.	Helps to sell the project to facilities. Increases likelihood of incorporation into sustainable practice. The stepped-wedge design can assess secular trends.
Identifying the population of facilities to target	Slower-to-adopt facilities are of concern.	Working with this population avoids ceiling effects (high performers) and insufficient commitment to change (laggards).
Site recruiting via operational structures	Hierarchical communications and reporting structure enhance facility identification and endorsement of program.	Provides access beyond “usual suspect” volunteer facilities and “friends of friends” facilities to enhance external validity.
Intervention and design		
Need for all participating sites to receive implementation support	Harder to justify the project on policy level if not all sites receive support. Can be a site recruiting tool.	Stepped wedge can accommodate this, though analysis is more complicated than traditional parallel-groups design Additionally, stepped wedge can enhance formative evaluation and evaluate secular trends.
Balance in randomization	Experience-based expertise contributes identifying characteristics relevant to success.	Sophisticated statistical expertise provides site alancing techniques.
Control condition	Sites seek as much active support as possible, as soon as possible.	Researchers develop a credible contrast condition by which to evaluate the impact of the implementation strategy.
Length of implementation support	Experience-based expertise suggests one year of support needed.	Pilot data agree, but the need for timely data provision requires steps in wedge of 4 rather than 12 months.
Need to work with existing VAMC staff without external research-funded support besides external facilitators	Resource limitations preclude deploying additional clinical or administrative staff (limitation of both OMHO and QUERI funding).	Makes sustainability more likely. Provides distinct scientific contribution enhancing effectiveness data beyond that from more traditional CCM clinical trials to date.
Delineating the interface between quality improvement program evaluation and research	The BHIP initiative is nationwide in scope and facility participation is not optional. However, a facility’s participation in this implementation project is optional.	Medical center participation in the project is the decision of the medical center director and mental health leadership, not individual provider. However, providers can choose not to participate in qualitative interviews. Patients can choose not to participate in health status and perception of care assessments.
Use of videoconference and telephone as main modalities for external facilitation	Budget (OMHO or QUERI) will not support frequent site visits by external facilitators.	Provides greater likelihood of spread of intervention strategy if successful.
Outcome assessment and analysis		
Identification of outcome domains and appropriate instruments	Program fidelity measures must be streamlined and targeted, and wherever possible benchmarked against national data.	Patient-level measures must be psychometrically valid and feasible in a heterogeneous patient population.
Both quality and health status impacts are important	Operational priority issues are (a) whether CCM can be implemented into BHIP teams and (b) whether CCM-guided BHIP teams have impact on the target population.	Hybrid type III designs can accommodate implementation outcomes and health status outcomes.
Data must both be scientifically valid and reported in a time frame useful to operational partners.	Three-year outcomes can help plan strategy for next initiatives, but are too late to make tactical improvements to this phase of BHIP roll-out.	Design and analysis accommodate “early looks” at the data on semi-annual basis, using adjustment of significance testing parameters.
Ethical and regulatory issues	A non-voluntary national initiative receives expert support from researchers in order to optimize their roll-out based on valid empirical data.	Researchers gather a broader range of data to answer relevant research questions from voluntary subjects.

Conceptual organization adapted from Bauer et al. [69]

Several overarching themes in establishing partner-based evaluation projects [64, 65] can also be highlighted. First, it is the priorities of the operational partners that make this type of evaluation project possible. These include not only the relative importance of the initiative but also the tangible resources and limitations that impact the project. An example of this is also found in the DIAMOND project [66], which was made possible not only by a shared sense of importance of improving depression treatment in Minnesota but also by the removal of fiscal barriers to establishing CCM-based procedures in a fee-for-service system [67, 68].

Second, an appreciation by all partners for the distinct skillsets each brings to the implementation process is essential. Related to this, at the sustainability and spread stage, both groups of partners must carefully knit their perspectives together to form a cohesive, consistent message in producing materials to guide the field—all of which must be articulated with the audience of end-users in mind.

Third, there must be a realistic appreciation of the distinct business cases for the operational and academic success the partners work within. For operational partners, this often requires measurable impacts on performance over relatively short time frame, while for researchers, academic productivity is assessed in terms of publications and presentations over a longer time horizon. Overall, the level of collaboration must go beyond a nodding appreciation to a willingness to incorporate diverse perspectives into the products, in the service of the highest quality, most feasible, most relevant project attainable.

## Abbreviations

BHIP: Behavioral Health Interdisciplinary Program
CCM: chronic care model
GLM: general linear model
i-PARIHS: Integrated Promoting Action Research on Implementation in Health Services
MCS: mental component score
OMHO: VA Office of Mental Health Operations
PACIC: Patient Assessment of Chronic Illness Care
PCS: physical component score
QUERI: VA Quality Enhancement Research Initiative
REP-F: Replicating Effective Programs
VA: Department of Veterans Affairs
VISN: Veterans Integrated Service Network
